# Reduced survival and reproductive success generates selection pressure for the dengue mosquito *Aedes aegypti* to evolve resistance against infection by the microsporidian parasite *Vavraia culicis*

**DOI:** 10.1111/eva.12144

**Published:** 2014-02-07

**Authors:** Victoria E Sy, Philip Agnew, Christine Sidobre, Yannis Michalakis

**Affiliations:** 1 Maladies Infectieuses et Vecteurs: Ecologie Génétique Evolution et Contrôle (MIVEGEC), CNRS UMR 5290Montpellier, France; 2 Instituto de Limnología “Dr. Raúl A. Ringuelet”, Universidad Nacional de La Plata-CONICETLa Plata, Buenos Aires, Argentina

**Keywords:** *Aedes aegypti*, *Beauveria bassiana*, evolution of resistance, insecticide, *Metarhizium anisopliae*, mosquito control, *Vavraia culicis*, virulence

## Abstract

The success and sustainability of control measures aimed at reducing the transmission of mosquito-borne diseases will depend on how they influence the fitness of mosquitoes in targeted populations. We investigated the effects of the microsporidian parasite *Vavraia culicis* on the survival, blood-feeding behaviour and reproductive success of female *Aedes aegypti* mosquitoes, the main vector of dengue. Infection reduced survival to adulthood and increased adult female mosquito age-dependent mortality relative to uninfected individuals; this additional mortality was closely correlated with the number of parasite spores they harboured when they died. In the first gonotrophic cycle, infected females were less likely to blood-feed, took smaller meals when they did so, and developed fewer eggs than uninfected females. Even though the conditions of this laboratory study favoured minimal developmental times, the costs of infection were already being experienced by the time females reached an age at which they could first reproduce. These results suggest there will be selection pressure for mosquitoes to evolve resistance against this pathogen if it is used as an agent in a control program to reduce the transmission of mosquito-borne human diseases.

## Introduction

Female mosquitoes pose a substantial burden on human health due to the diseases they vector (World Health Organization 2010). Mosquito control is often the main means of restricting the transmission of these diseases (World Health Organization 2012). Many of these control measures reduce mosquito fitness and, in doing so, generate selection pressure for mosquitoes to evolve resistance against them (Georghiou 1972). For example, the application of chemical insecticides can decimate targeted populations and be an efficient means of mosquito control (Spielman and D'Antonio 2001). However, this imposes strong selection pressure for resistance to evolve against the chemicals used, and resistant mosquitoes are a widespread problem concerning all major vector species and all classes of insecticide (World Health Organization 2012). In principle, it should be possible to control the transmission of mosquito-borne diseases, without creating selection pressure for resistance to evolve, by control measures having a minimal impact on mosquito fitness. One way to do this is by measures taking into account differences in the age-dependent structure of when female mosquitoes reproduce and when they transmit disease (Cook et al. 2008; Read et al. 2009).

In general, the agents of human disease vectored by mosquitoes must complete a period of within-mosquito development before they can be transmitted. For example, the mean extrinsic incubation period (EIP) of *Plasmodium falciparum* has been estimated as ranging from 12 to 18 days in average temperatures of 24°C (Blanford et al. 2013), while that of the dengue virus varies from 10 to 20 days at 25°C (Chan and Johansson 2012). These incubation periods are often considerably longer than the time taken for female mosquitoes to complete a gonotrophic cycle (from blood-feeding, to oviposition, to blood-feeding again), which is usually in the order of a few days (Clements 1992). Furthermore, in natural conditions, relatively few female mosquitoes survive long enough to complete more than a couple of gonotrophic cycles, for example, the proportion of *Aedes aegypti* females surviving > 10 days was estimated as < 10% in Brazil (Maciel-De-Freitas et al. 2007). This means the females surviving long enough to vector human disease are already likely to have achieved most of their expected lifetime reproductive success. A consequence is that control measures specifically aimed at these reproductively successful females will generate much less selection pressure for them to evolve resistance than measures that also target younger females that have not achieved as large a proportion of their expected lifetime reproductive success.

The specific control of relatively old females could potentially be achieved by pathogens that infect mosquitoes. Many pathogens do not kill their hosts directly upon contact, but increase their morbidity and mortality with time postinfection. This delay in the expression of pathogen virulence (or cost to host fitness) can provide infected hosts with the opportunity to reproduce before the mounting costs of infection prohibit them from doing so. For example, daily rates of mosquito mortality increase dramatically as fungal sporozoites mature in the haemocoel of mosquitoes in the days following their exposure to spores of *Metarhizium anisopliae* or *Beauveria bassiana* (e.g. Blanford et al. 2005; Scholte et al. 2005). Infected females generally have time to complete at least one gonotrophic cycle before dying, with relatively few surviving long enough to vector human disease.

The microsporidian parasite *Vavraia culicis* infects mosquito larvae and increases the morbidity and mortality of its hosts with time since infection (Weiser 1980). The parasite's production of spores is usually detected from approximately 10 days postinfection, whether the host is still a larva or has reached adulthood (Bedhomme et al. 2004). The accumulation of spores can result in extensive damage to host tissues (Bano 1958) and is correlated with increased rates of host mortality (Agnew et al. 2004). Reynolds (1970) found *V. culicis* infections had little effect on *Culex quinquefasciatus* mosquitoes reaching adulthood, but reduced the longevity and lifetime reproductive success of adult females. Weiser (1980) found this was a general pattern for the costs experienced by mosquitoes infected by *V. culicis* when reviewing its potential as a biological control agent and concluded the reduced longevity of infected adult female *Anopheles* mosquitoes can prevent the transmission of malaria. More recent studies have reached a similar conclusion, particularly in the context of *V. culicis* as a control agent with late-in-life activity against mosquitoes (Koella et al. 2009a; Koella et al. 2009b; Lorenz and Koella 2011).

In this study, we investigated the costs of *V. culicis* infections on the survival and reproductive success of adult female *Ae. aegypti*, the main vector of dengue. We found infection reduced the survival of adult females. The increase in their daily rate of mortality, relative to uninfected females, was closely correlated with the number of spores they harboured when they died. Infected females were less likely to take a blood meal in the first few days following emergence. Those that did feed took smaller volumes of blood than uninfected females and developed fewer eggs. A model for the within-host dynamics of infection predicts the costs experienced by females will be greater and occur earlier relative to their age at first reproduction as larval growth rates slow. As rearing conditions in laboratory studies are often optimal for larval growth, such studies are likely to result in the costs of infection being underestimated relative to those experienced by infected mosquitoes in natural environments. This study suggests there will be selection pressure for mosquitoes to evolve resistance against *V. culicis* infections.

## Materials and methods

### Host and parasite material

*Aedes aegypti* (L.) is a highly anthropophilic mosquito distributed across the tropical and subtropical regions of the world. It is the main vector of the dengue virus, the causative agent of dengue and dengue haemorrhagic fever. The strain of *Ae. aegypti* used in this experiment was originally derived from a large number of eggs collected in Tingua, Brazil, and was kindly provided by Dr Ricardo Lourenço de Oliveira of the Instituto Oswaldo Cruz (Rio de Janeiro, Brazil). At the time of the experiments, it had been maintained in the laboratory for 35 generations with ∼ 3000 breeding adults per generation.

The type specimen for *V. culicis* was isolated from *C. pipiens* (Weiser 1946). The material used in this experiment derives from the Florida isolate of *V. culicis* (Vavra and Becnel 2007). It was originally isolated from *Ae. albopictus* during a survey, which also found infected *Ae. aegypti* in the same breeding sites (Fukuda et al. 1997). It was kindly provided by Dr J.J. Becnel of the United States Department of Agriculture (USDA), Gainesville. The material was originally amplified using lepidopteran hosts, but has more recently been maintained using *Ae. aegypti*. A full description of its development can be found in Vavra and Becnel (2007), and details of its interaction with *Ae. aegypti* are reviewed in Michalakis et al. (2008).

### Experimental procedure

Two separate experiments investigated the effects of *V. culicis* on life-history traits of adult female *Ae. aegypti*. The first experiment recorded the effects of infection on the survival, blood-feeding success and fecundity of adult females. The second compared the effects of infection on the survival of adult females provided with only sugar, a single blood meal or blood meals at weekly intervals. Female survival was followed for 3 weeks, a period within which most disease transmission is likely to occur in natural conditions. Both experiments were conducted in the same climate-controlled room maintained at a temperature of 25°C ± 1°C and relative humidity of 75% ± 5% (means ± SD, respectively) with a 12:12 h light:dark cycle.

### Experiment I

This experiment involved 14 cages (30 × 34 × 42 cm). Each cage initially contained 75 adult mosquitoes; 50 females (25 infected, 25 uninfected) and 25 males. Eight cages were used to assess adult survival; males were uninfected in these cages. Six cages were used to assess female blood-feeding behaviour (probability of taking a meal, volume of blood taken) and their fecundity; to test for the effect of male infection on these traits, three cages had infected males and three cages had uninfected males.

To begin Experiment I, several thousand eggs were immersed in mineral water (Eau de Source, Carrefour, France) and simultaneously hatched under reduced atmospheric pressure. Groups of 60 larvae were transferred to Petri dishes (diam. 55 mm) containing 10 ml of mineral water and 3.6 mg of fish food (Tetramin MicroFood, Melle, Germany). A total of 42 Petri dishes were prepared; three for each adult cage.

Larvae were exposed to infection by adding 2.4 × 10^6^ *V. culicis* spores in 1 mL of mineral water to 17 Petri dishes, giving a concentration of 4.0 × 10^5^ spores/larva. A matching volume of mineral water was added to Petri dishes not receiving spores. Dishes were kept in a climate-controlled chamber at 27°C. Exposure to infection was stopped 24 h later by rinsing larvae and transferring them to new Petri dishes containing clean water. Following Bedhomme et al. (2004), these conditions should have yielded an infection success of > 95%, which was found to be the case; thus, hereafter, we refer to them as ‘infected’ individuals.

Two Petri dishes containing uninfected larvae and one dish containing infected larvae were assigned to each of the 14 adult cages, except for the three cages requiring infected males, which were assigned two dishes containing infected larvae and one dish containing uninfected larvae. Larvae completed their development after being transferred to individual *Drosophila* vials (diam. 20 × 95 mm) containing 5 ml of mineral water and 2.0 mg of fish food. Two racks holding a total of 80 tubes of uninfected larvae and two racks holding 60 tubes of infected larvae were prepared for each cage and physically placed together to form a block, except for the three cages destined to hold infected males where two racks holding a total of 80 infected larvae and two racks holding 60 tubes of uninfected larvae were prepared. Tubes were followed on a daily basis, and larval mortality or pupation was recorded. In the event of pupation, the tube was sealed with a foam bung. Sex and day of emergence were recorded for adults.

Adults were added to the cage corresponding with their rack on their day of emergence until cages held the desired number and sex of infected and uninfected individuals. In a few cases, it was not possible to complete a cage using only tubes with which it had been associated (e.g. due to larval mortality or an unfavourable sex-ratio) and surplus adults from tubes in other blocks were taken to complete a cage. Most of the females (94%) put in cages were 9–11 days old when they emerged, the remaining females were either 12 or 13 days old.

Twenty-four hours after the last adults were added to cages, an anesthetised female OF1-ICR mouse was placed on each cage between 10–11 h and from 14 to 15 h on the same day. A different mouse was used for each cage, with the same individual being used for both feeding periods. Cages contained a wicked bottle of 10% sugar solution, which was removed from cages approximately 16 h prior to the first opportunity to blood-feed and returned after the second opportunity had finished. Individuals dying during the period of sugar removal were removed and not included in subsequent analyses as their death was considered as an artefact of the experimental design. Sugar solutions were replaced weekly.

### Effect of infection on longevity

The eight cages used to measure adult longevity were monitored daily. Dead individuals were transferred to numbered 1.5-mL plastic vials and stored at −20°C until checked for infection and, if infected, to quantify the number of *V. culicis* spores. All individuals alive 3 weeks after the first blood meal were killed by exposure to CO_2_ and treated in the same way.

Cages were provided with a 250-mL plastic pot half-filled with water and lined with filter paper for oviposition. These pots were introduced into cages 2 days after blood-feeding and remained in place for the duration of the experiment, being refilled with water when necessary.

### Effect of infection on blood-feeding behaviour and fecundity

The six cages used to measure these traits were monitored daily as above, but females were isolated for oviposition the day after the opportunity to blood-feed. They were isolated in standard *Drosophila* vials sealed with a foam bung. A 1.5-mL plastic vial containing 0.75 mL of water was placed within each tube for oviposition. Vials were monitored daily for mortality and oviposition. Females still alive 11 days after blood-feeding were killed. Dead individuals were kept at −20°C until further analysis.

The number of eggs each female laid was counted using a dissecting microscope. The amount of blood they took was estimated indirectly by determining the amount of haematin in excreta present in a vial (Briegel 1980). Females in vials without black pellets, characteristic of excreta from digested blood, were assumed not to have blood-fed as excreta were always present in vials where females laid eggs and autogeny is unknown for *Ae. aegypti*. Females laying less than 75 eggs were dissected to check for retained eggs. We judged the presence of retained eggs as an artefact arising from females being isolated in individual vials without access to a source of sugar, such that these eggs would have been laid had sugar been available. Consequently, a female's fecundity was determined as the total number of laid and retained eggs.

### Effect of infection on adult size and correlated traits

Adult size is an important trait that can influence blood meal size, fecundity and longevity (Clements 1992); it can also be influenced by infection. Adult female size was estimated by measuring wing length using a dissecting microscope fitted with a graduated eye-piece (precision: 0.03 mm). Both wings were removed from each individual and measured from the alular notch to the distal extreme, excluding the fringe scales. All females involved in fecundity estimates were measured, and their size was taken into account when analysing the effect of infection on blood-feeding and fecundity.

### Experiment II

#### Nutritional regime and longevity

This experiment was similar to Experiment I but assessed female longevity as a function of different opportunities to blood-feed. Adults were arranged in 16 cages, each containing 50 females (25 infected and 25 uninfected) and 25 uninfected males. Females in eight cages were provided with the opportunity to blood-feed on a single day (as in Experiment I), while females in five cages were given the opportunity to blood-feed 1 day a week for 3 weeks, and females in three remaining cages were not given the opportunity to blood-feed at all.

Larvae were reared and assigned to adult cages using the same protocol as in Experiment I. However, the number of infected larvae individually reared for each cage was raised to 80 to ensure each block of larval tubes yielded at least 25 infected adult females. The majority of females (97%) added to cages were between 9 and 12 days old when they emerged, the remaining females emerged when 13 days old.

Once all adult cages were complete, sugar solutions were removed. The following day, two blood meals were offered to cages as in Experiment I, except for three cages where females were not offered the opportunity to blood-feed (the ‘sugar-only’ treatment). As in Experiment I, sugar solutions were returned to cages after the second blood meal. This feeding procedure, including sugar removal, was repeated every week for the five cages forming the ‘multiple blood meal’ treatment. Where possible, the same mouse was used for the same cage in successive weeks. This was not always possible due to the death of two mice. These mice were replaced with other individuals from the same colony and of the same age. All cages were provided with an oviposition pot 2 days after the first blood meal was offered; these were replaced weekly in cages of the multiple blood meal treatment.

Not all female mosquitoes offered the opportunity to blood-feed took a meal. As the aim of the second experiment was to test whether having one or more blood meals influenced a female's survival, females that had not blood-fed were removed from cages the day after they had been presented with a mouse. Non-blood-fed females were visually identified by the colour and size of their abdomen. The presence or absence of blood was later verified by the colour of the homogenate when females were prepared for spore counting.

As in Experiment I, mortality was followed for 3 weeks following the first opportunity to blood-feed. Dead individuals were collected daily, stored in individually numbered plastic 1.5-mL vials and stored at −20°C until further treatment. Wing lengths were not measured for females in Experiment II.

#### Identifying infections

To identify infected females, each individual was homogenized in a 1.5-mL plastic vial containing 0.5 mL of demineralised water. Spores were counted using a haemocytometer and phase-contrast light microscope; each spore observed equated to 5000 spores in the mosquito. If no spores were seen, the individual was assumed not to have been infected. When spore counts were >3, the individual was considered infected. A few individuals with counts of 1 ≤ 3 spores were discarded to avoid including false positives; most infections were unambiguous. Furthermore, the number of infected and uninfected adults estimated by the presence/absence of spores agreed closely with the expected number of infected/uninfected individuals from each cage. This indicates almost all larvae exposed to infection became infected.

### Statistical analyses

Adult female survival was estimated with log-likelihood models based on the Weibull survival function,


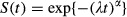


where survival (*S*) to *t* days after the first opportunity to blood-feed is determined by the shape and scale parameters *α* and *λ*, respectively.

Models took into account when females died or were censored. Censored individuals were those still alive at the end of each experiment or removed alive from cages in Experiment II when judged to not have blood-fed when given the opportunity to do so the previous day. Estimates of *α* and *λ* varied among models due to the addition/subtraction of values associated with the individual or combined effects of experiment, infection status, treatment and cage of origin. Models were compared using Akaike's Information Criteria (AIC) weighted for differences among models. These included models where *α* was set to 1.0, such that survival time was exponentially distributed with a constant hazard function of *λ*. Models were fitted using the nonlinear platform of JMP® version 9.0.2. Full details are described in Appendix S1.

The probability females would take a blood meal in the six cages of Experiment I was analysed with a binomial split-plot model using the *lme4* package of R (http://r-forge.r-project.org/projects/lme4/). Whole plots were cages where males were either infected or uninfected, with the subplot being female infection status within cages. The volume of blood taken by females in these cages was analysed in the same split-plot design with a mixed model analysis of variance (anova) using JMP; this model was also used to analyse female fecundity.

The effect of infection on the size of adult females in Experiment I was analysed with a split-plot model taking cage as a random effect. This model did not take male infection status into account as it could have no influence on female size. The models for blood meal volume and fecundity were also performed using wing length as a covariate. In the analysis of the fecundity of blood-fed females, only those with a total of more than 33 eggs (laid or retained) were included to normalise the data's distribution.

## Results

### Pre-adult mortality

The majority of larvae emerged as adults; however, in both experiments, the probability of emerging was lower for individuals exposed to *V. culicis*; probability of emergence for uninfected vs. infected larvae; Experiment I, 86.8% vs 80.5%; Experiment II, 91.5% vs 71.9%, respectively.

### Infection reduced adult female survival

Infected adult females were more than twice as likely to die than uninfected females (46% vs 18%) in the 3 weeks following the opportunity to blood-feed in both Experiments I and II: infected females dying, 56 of 122 and 123 of 268; uninfected females dying, 31 of 174 and 55 of 311, respectively.

### Infection induced age-dependent mortality

**Figure 1:**
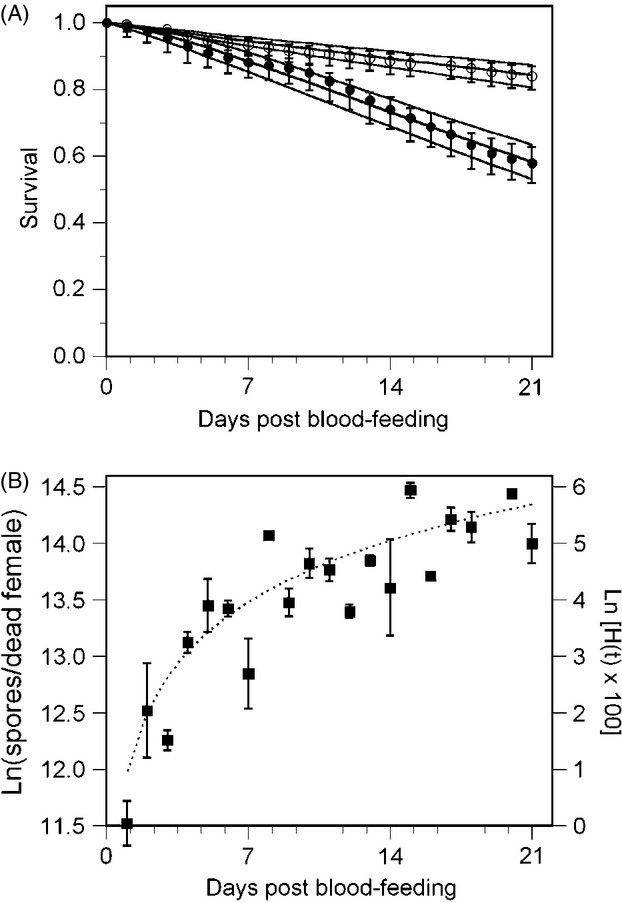
(A) Survival of uninfected (open symbols) and infected (closed symbols) female mosquitoes in the 3 weeks following the first opportunity to blood-feed (± nonparametric 95% confidence intervals) for the observed data. The black lines represent the best-fit survival curves for uninfected females, *S*(*t*) = exp[-(0.008*t*)], and infected females, *S*(*t*) = exp[−([0.030*t*]^1.345^)]. (B) The symbols represent the natural logarithm of the mean number of spores (± SE) recovered from dead females during the 3 weeks following the first opportunity to blood-feed. The dotted line represents the natural logarithm of the cumulative risk of additional mortality experienced by infected females; *H*(*t*) = (0.025*t*)^1.571^ in the same period. These figures do not include data from the ‘sugar-only’ treatment of Experiment II.

The observed and estimated survival of females from Experiment I and those of the ‘single blood meal’ and ‘multiple blood meal’ treatments of Experiment II are shown in Fig. 1A; these data do not include females from the ‘sugar-only’ treatment of Experiment II as their rate of mortality was found to be different.

The survival of uninfected adult females (*S*_*uninf*_) was best described with a constant rate of mortality


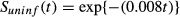


whereas the best model describing the survival of infected females (*S*_*inf*_) found their rate of mortality to increase over time,


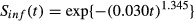


and thus with their age.

Models were not significantly improved by allowing *λ* and/or *α* to vary with experiment, interactions between experiment and infection, or replicate cages within experiments (see Appendix S1 for full details).

The survival of infected females was also estimated as,


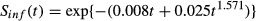


where the first term within the inner brackets describes the common background mortality experienced by infected and uninfected females (e.g. due to their shared environment and age), while the second term describes the additional mortality of infected females due to infection. This latter term estimates the relative survival of infected females due to infection (Dickman et al. 2004).

### Increased mortality of infected females correlated with spore production

The cumulative risk of mortality due to infection, *H*_*inf*_(*t*), estimated from the relative survival of infected females,


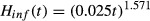


was closely correlated with the average number of spores harboured by infected females at the time of their death; correlation *ln*[mean number of spores(*t*)] and *ln*[(0.25*t*)^1.571^], *r *=* *0.883 (Fig. 1B). This suggests a causal relationship between *V. culicis*’ spore production and the increased mortality of infected females.

### Blood-feeding improved female survival

The results presented above do not include females from the ‘sugar-only’ treatment in Experiment II as the best-fit model for survival found their mortality was greater than that of females in the two treatments offered blood. This difference, however, did not depend on a female's infection status, or an interaction between feeding and infection treatments (see Appendix S1).

### Infection reduced blood-feeding and fecundity

**Figure 2:**
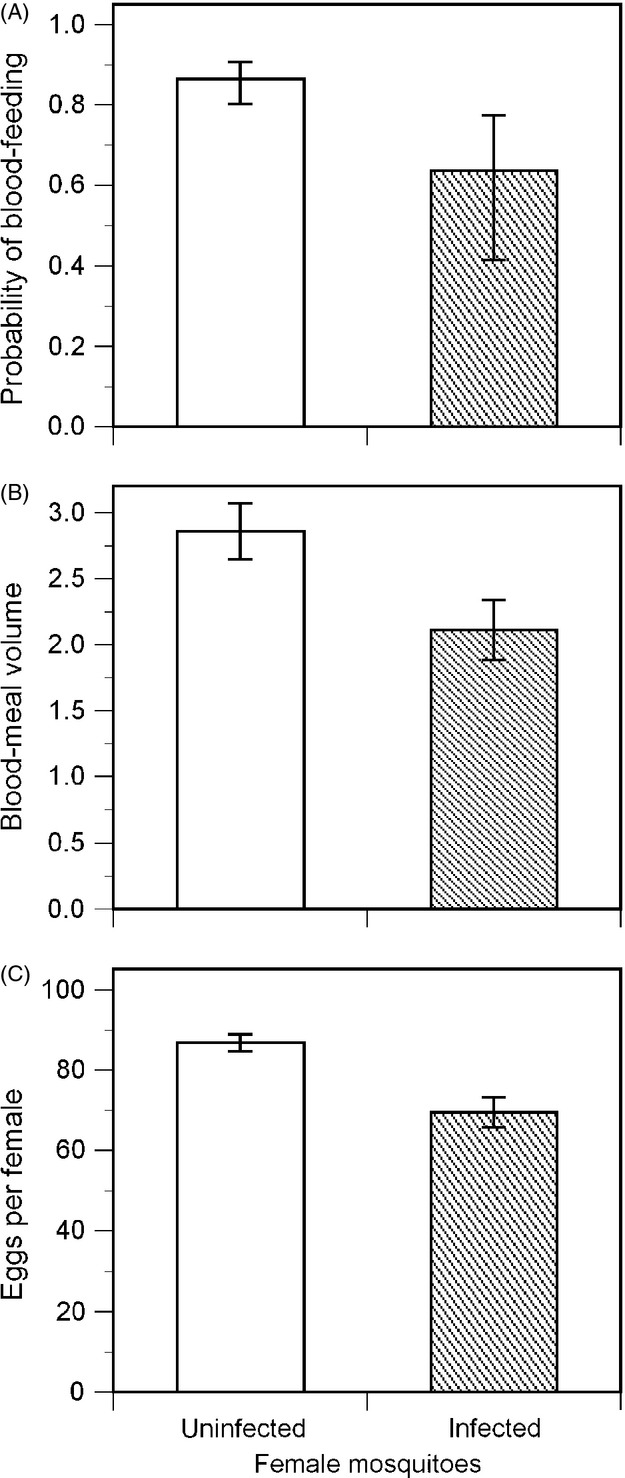
The blood-feeding behaviour and fecundity of uninfected (open columns) and infected (filled columns) females from the ‘fecundity’ cages of Experiment I. (A) The probability that females would take a blood meal (± SE). (B) The estimated volume (*μ*L) of blood taken by females that blood-fed (± SE). (C) The number of eggs produced by females that took blood meals (± SE).

Infected females were significantly less likely to take a blood meal than uninfected females (Experiment I; *Z *=* *2.070, *P *=* *0.039, *N* = 251). Infection reduced the probability of feeding by approximately 25% relative to uninfected females (Fig. 2A).

For those females that did blood-feed, the size of the blood meal taken by infected females was significantly smaller than that of uninfected females (*F*_1,5_ = 25.930, *P *=* *0.003, *N* = 182) (Fig. 2B).

When only females taking a blood meal were considered, the total number of eggs developed by infected females was less than that of uninfected females by approximately 20%; 70 vs 87, respectively; *F*_1,4_ = 84.912, *P *=* *0.001, *N* = 167 (Fig. 2C).

Traits related to blood-feeding and fecundity are likely to be correlated with adult female size (Clements 1992). There was an effect of infection on size as infected females had shorter wings than uninfected females (2.45 vs 2.58 mm; *F*_1,5_ = 86.465; *P *<* *0.001; *N* = 267). Hence, the results above could be due to an indirect effect of infection on adult size, rather than a direct consequence of infection *per se*. To assess this, blood meal size and fecundity were re-analysed with anova models including wing length as a covariate. These analyses found larger females took larger meals (*F*_1,176_ = 5.306; *P *=* *0.022; *N* = 182), but the effect of infection remained with infected females taking significantly smaller blood meals (*F*_1,12_ = 6.636; *P *=* *0.024; *N* = 182). The effect of infection on fecundity also remained significant when female size was taken into account (*F*_1,10_ = 14.494; *P *=* *0.004; *N* = 167). Hence, although some of the negative effects of *V. culicis* on these traits can be attributed to an indirect effect of infection on female size, there were still direct negative effects of infection on the blood-feeding behaviour and fecundity of infected females.

### Effect of male infection on female traits

There was no significant effect of male infection on a female's probability of blood-feeding (*Z *=* *1.724, *P *=* *0.085, *N* = 251); the size of meals taken; (*F*_1,4_ = 0.280, *P *=* *0.623, *N* = 182) or their fecundity (*F*_1,4_ = 0.452, *P *=* *0.539, *N* = 167).

## Discussion

In this study, the majority of mosquitoes infected by *V. culicis* survived to adulthood (70–80%). Infection induced age-dependent mortality in adult females and reduced their survival relative to uninfected females (Fig. 1A). The fecundity of infected females in their first gonotrophic cycle was also reduced, with them developing approximately 20% fewer eggs than uninfected females (Fig. 2C). These results agree with the general pattern of costs experienced by other species of mosquito infected with *V. culicis* as identified by Weiser (Weiser 1980). To this, we can add that the additional mortality experienced by adult females infected with *V. culicis* was closely correlated with the number of spores they harboured at the time of their death. Infected females were less likely to take a blood meal in the first few days after emergence than uninfected females (Fig. 2A) and took smaller volumes of blood when they did feed (Fig. 2B). We discuss these results in relation to the reproductive success and selection pressure experienced by females when infected by *V. culicis*, as well as the pathogen's potential influence on their capacity to vector human disease.

### Effect of infection on reproductive success

The fitness costs experienced by mosquitoes targeted in a control program will determine the strength of selection for them to evolve resistance against the control measure. The delay until resistance begins to evolve, as well as the rate and magnitude of its evolution will be proportional to the selection pressures involved (Georghiou 1972). A measure of the strength of selection can be estimated for control measures involving pathogens by comparing the relative fitness of infected and uninfected mosquitoes, such that


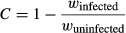


where *C* is the coefficient of selection for resistance to evolve and *w* is mosquito fitness.

Our data are not sufficient to estimate the overall fitness of either infected or uninfected mosquitoes, but provide information on some important components of fitness. For example, infected mosquitoes were less likely to survive to adulthood. The costs associated with this reduced survival are maximal as the individuals concerned will never reproduce. Infection also reduced the probability of females taking blood when presented with two one-hour-long opportunities to feed on an anaesthetised mouse. The costs of not blood-feeding are also maximal as *Ae. aegypti* females cannot mature a clutch of eggs without blood-feeding as an adult (Christophers 1960). However, we assume unfed females would have taken blood if our experimental protocol had given them further opportunities to do so. In which case, our results suggest infected females will take longer to initiate their first gonotrophic cycle than uninfected females, with the cost being increased exposure to adult mortality before reproducing. When infected females did feed in this study, they took smaller volumes of blood than uninfected females and developed fewer eggs. Infected females could potentially compensate for this by taking additional blood meals per gonotrophic cycle; this was not possible beyond the two feeding opportunities offered to females in Experiment I. The benefits of feeding more frequently, however, need to be offset against the risk of being killed at each feeding event (Edman and Scott 1987).

Our results found no evidence of *V. culicis* infections reducing the reproductive success of adult male mosquitoes as females housed with infected males were just as likely to lay eggs and had similar fecundities to those housed with uninfected males. This could be because male reproductive success was relatively less exposed to the time-dependent costs of *V. culicis* infections. For example, male investment in spermatogenesis is all but complete within 24 h of their emergence as adults (Clements 1992), whereas females can only mature a first clutch of eggs after having taken a blood meal as an adult. The number of unfertilised and sexually receptive adult females will diminish rapidly in caged environments, such as this study, thus condensing most male mating success into a short period following the introduction of females into adult cages. The reproductive success of infected males might have been less had they been in competition for females with uninfected males, especially if mate-seeking activity depended on metabolic resources carried over from larval life (c.f. Rivero et al. 2007), but this was not the case in this study.

Despite the fitness costs identified above, most mosquitoes infected with *V. culicis* survived to adulthood and lived long enough to reproduce at least once in the conditions of our experiments. These results suggest there will be selection pressure for mosquitoes to evolve resistance against *V. culicis* infections, but this pressure is likely to be weaker than that generated by some of the more widely used means of mosquito control and weaker for males rather than females.

### Effect of developmental time on selection pressure

The increased rate of adult mortality for infected females was closely correlated with the number of spores they harboured at the time of their death (Fig. 1B). This suggests *V. culicis*’ spore production can be used as a proximate measure of the costs experienced by its host. In this section, we focus on how environmental factors may influence the duration of mosquito development, and consequently their reproductive schedule, relative to the parasite's spore production.

The time required for larval mosquitoes to complete their development is influenced by many environmental variables, including temperature, larval density, food availability, pollution and interspecific competition (Clements 1992). The larvae in this study were reared in conditions favouring a minimal period of development, with most females emerging by 10–11 days posthatching. These conditions do not reflect those encountered by most mosquitoes in natural environments where development times are often longer. For example, the development of *Ae. aegypti* larvae has been reported as 14–17 days for natural breeding sites in Thailand (Southwood et al. 1972) and varying from 11 to 28 days across different sites in Australia (Tun-Lin et al. 2000). Differences in mean temperatures or diurnal range of temperature variation of natural and laboratory environments will account for some of this variation. Another important factor will be larval food availability. In laboratory environments, food is often abundant and there is relatively little density-dependent competition among larvae for it. In contrast, the amount of food available in natural sites is often limiting, and greater larval densities mean there is more intense competition among larvae for it (e.g. Subra and Mouchet 1984; Arrivillaga and Barrera 2004). When food availability is sufficiently limiting, growth ceases and larvae maintain themselves with what little food is available or by metabolising stored energetic resources until more food become available. In such conditions, larval developmental times can be considerably longer, for example, the duration of the fourth larval instar of *Ae. aegypti* can be prolonged almost indefinitely when larvae are not provided with enough nutritional resources to initiate pupation (Gilpin and McClelland 1979).

The factors influencing microsporidian development are less known. Some environmental variables are likely to influence their development in a manner similar to that of their hosts. For example, the development of larvae and their infections will broadly be positively correlated with ambient water temperatures. However, each organism may tolerate a different range of temperatures and be more or less sensitive to departures from the particular temperature at which their development is optimal (e.g. Becnel and Undeen 1992). However, as all microsporidia are obligate intracellular parasites, their development is likely to depend more directly on conditions within the individual host cell they occupy, rather than the environmental conditions experienced by their host. Furthermore, the physiological mechanisms ensuring host cell homeostasis in face of variable environmental conditions are likely to buffer microsporidian growth against such change. For example, *V. culicis* growth and spore production within host cells are maintained when low food availability in the aquatic environment brings larval development to a halt (Bedhomme et al. 2004). Spores are produced at a slower rate in such conditions, indicating microsporidian growth is limited by host cell resource availability to some extent (Bedhomme et al. 2004). We expect limiting environmental food availability will have a much greater effect in prolonging larval developmental times relative to its influence on microsporidian developmental times.

**Figure 3:**
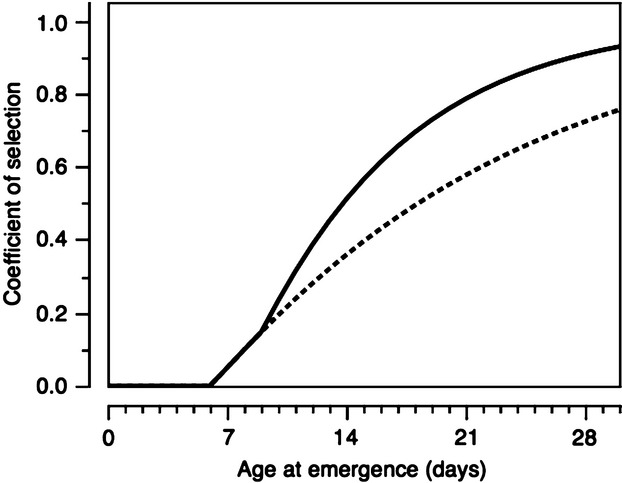
Predictions for the coefficient of selection against *Vavraia culicis*-infected female mosquitoes reaching first reproduction (emergence + 3 days) as a function of age at emergence. The dashed line shows the estimate based on the survival of adults, the solid line includes pre-adult mortality. Values used; *λ*_*sp*_ = 1.0, *α*_*sp*_ = 1.05, *c *=* *10, *k*_*1*_ = 0.05, *k*_*2*_ = 0.05 (see Appendix S2).

Figure 3 shows estimates for the coefficient of selection against infected female mosquitoes surviving to 3 days postemergence (approximately the age at first reproduction) as a function of larval development times when their relative survival is reduced by infection from 10 days posthatching. The dotted line traces the coefficient of selection when only adult mortality is observed, while the solid line takes into account the pre-adult mortality of larvae and pupae. Full details of the model generating these estimates are given in Appendix S2. The estimates suggest studies such as this and Lorenz and Koella (2011) will tend to be biased towards low estimates for the strength of selection against infected females as larvae developed in environmental conditions favouring minimal larval developmental times. In the latter study, larvae were already 48 h old before being exposed to infection, thus further reducing their exposure to the costs of infection prior to emergence or first reproduction. In contrast, the strength of selection against infected *C. quinquefasciatus* reaching adulthood was much greater in another study where limited daily supplies of food extended larval developmental times beyond the onset of *V. culicis*’ spore production (Agnew et al. 2004).

The predictions above suggest larval development times will be influential in determining the strength of selection against mosquitoes infected by *V. culicis*. The same arguments may apply to other pathogens whose costs increase with time since infection and can be used to infect mosquito larvae, such as the entomopathogenic fungi (e.g. Pereira et al. 2009; Kamalakannan and Murugan 2011). If mosquito larvae are to be the targets for infection in a control program, laboratory studies with the pathogen should explore the host–pathogen interaction across a range of larval developmental times so as to avoid underestimating the strength of selection for resistance for mosquitoes to evolve against the pathogen concerned.

### Vectorial capacity of infected females

Our results broadly suggest the capacity of *Ae. aegypti* to vector human disease will be reduced by *V. culicis* as infected mosquitoes were less likely to survive to adulthood and adult females experienced increased age-dependent rates of mortality relative to uninfected females.

We do not extensively discuss the influence of *V. culicis* on the capacity of its female hosts to vector human disease as we have no data on the subject. Furthermore, without data it is not clear as to how *V. culicis*’ effects on traits other than mosquito survival will influence their host's capacity to vector disease, for example, opposing results have been reported for the effect of adult size on the ability of *Ae. aegypti* females to vector the dengue virus (Sumanochitrapon et al. 1998; Alto et al. 2008). Studies involving mosquitoes infected with pathogens and agents of human disease are needed to assess the overall influence of a pathogen on its host's capacity to vector disease (e.g. Garza-Hernandez et al. 2013). We do, however, expand our discussion concerning the altered blood-feeding behaviour of females infected with *V. culicis* as blood-feeding behaviours strongly influence the epidemiology of vector-borne diseases (Macdonald 1957).

The altered blood-feeding behaviour of females infected with *V. culicis* is likely to influence their capacity as vectors. Predictions for whether there will be a relative increase or decrease in the number of infectious bites from females infected with *V. culicis* depend on how a female's biting rate and the number of meals taken per gonotrophic cycle are assumed to interact (Cator et al. 2012). For example, if females infected with *V. culicis* are assumed to take more blood meals per gonotrophic cycle than uninfected females (to compensate for smaller volumes of each meal), this can be expected, (i) to increase the probability of them contracting a blood-borne agent of human disease and subsequently transmitting it when they become competent to do so; and (ii) to decrease the probability of them surviving due to the risk of being killed at each feeding event (Edman and Scott 1987). If females infected with *V. culicis* have a lower daily biting rate than uninfected females, this can be expected, (i) to increase the time for them to complete a gonotrophic cycle and thus increase the probability of them dying before completing the cycle; and (ii) to reduce the number of gonotrophic cycles completed in the time, it takes a human pathogen to complete its EIP. This latter point will interact with the second point above as infected females will be less exposed to feeding-related mortality if they complete fewer gonotrophic cycles in the period before they can vector disease.

It should also be taken into account that the probability of a female contracting a blood-borne agent of human disease depends on the prevalence of infection in the human population. This can be calculated as, 1 − (1 − *a*)^*b*^ where *a* is prevalence of infection in the human population and *b* is the number of blood meals taken. When the prevalence of infection is high, a female's exposure to blood-borne pathogens is also high and increasing the number of bites taken per gonotrophic cycle will have relatively little influence on the probability a female contracts an agent of human disease. When the prevalence of disease is low in the human population, the probability a female contracts an agent of human disease increases to a greater extent as the number of meals she takes increases. However, the overall probability of taking an infected blood meal remains low.

We estimated the potential influence of altered blood-feeding behaviour and reduced survival on the relative probability of an adult female mosquito infected with *V. culicis* would contract a human pathogen in her first gonotrophic cycle and survive a period equal to the EIP of the dengue virus using data from this study (see Appendix S3 for full details). This probability generally increased as the prevalence of dengue in the human population decreased and as the probability of females surviving a blood-feeding event increased. When both conditions are satisfied, females infected with *V. culicis* are estimated as potentially being relatively better vectors of dengue than those not infected with *V. culicis*. In most conditions, however, they were not. These estimates should not be interpreted literally, especially as they depend on untested assumptions relating the volume of blood taken per individual feeding event to the number of meals needed to complete a gonotrophic cycle. However, these estimates indicate that reductions in the capacity of female mosquitoes to vector disease due to a pathogen increasing their age-dependent mortality can be countered (or even reversed) by relatively modest changes in their blood-feeding behaviour (c.f. Cator et al. 2012). This topic is worth further investigation as other pathogens with late-in-life activity against mosquitoes are also reported to alter the blood-feeding behaviour of female mosquitoes (e.g. Scholte et al. 2006; Blanford et al. 2011; Darbro et al. 2012).

Finally, we note that the influence of *V. culicis* on the epidemiology of mosquito-borne diseases will depend on the proportion of mosquitoes infected by both *V. culicis* and an agent of human disease, whereas all females infected by *V. culicis* are likely to experience selection pressure to evolve resistance against its infection. Thus, the duration of any epidemiological benefits in a control program may be more strongly influenced by evolutionary pressures on the nonvectoring proportion of female mosquitoes in targeted populations, rather than the proportion capable of vectoring human disease.

## Conclusion

The results of this study find *V. culicis* infections reduce the reproductive success of female *Ae. aegypti* mosquitoes and do so in a manner reported for other mosquitoes. These costs will favour mosquitoes to evolve resistance against *V. culicis* infections. We estimate the altered blood-feeding behaviour of females infected with *V. culicis* will probably reduce their reproductive success and their capacity to vector disease. However, an increase in the capacity of *V. culicis*-infected females to vector disease could arise in conditions where the prevalence of infection in the human population is low. We argue the costs female mosquitoes experience prior to first reproduction will increase as larval developmental times increase and laboratory studies favouring rapid larval development will underestimate such costs. A similar scenario is likely for other pathogens used to infect larvae and whose costs increase with time since infection.

There is no direct evidence of how mosquitoes evolve in response to *V. culicis* or other microsporidian infections. It has been suggested they could alter their life-history traits to bring forward their schedule of reproduction (Koella et al. 2009a,b2009b). Alternatively, they could try and suppress infection (e.g. Rost-Roszkowska et al. 2013) or evolve tolerance to its costs, as reported for other invertebrates repeatedly exposed to microsporidian infection (Zbinden et al. 2008; Vijendravarma et al. 2009). Without further data, it is not possible to predict how mosquitoes will evolve in response to *V. culicis* infections and the consequences this could have on their capacity to vector human disease. The only clear prediction currently possible is that *V. culicis* infections generate selection pressure for mosquitoes to evolve resistance against them.

## Data archiving statement

Data for this study are available from the Dryad Digital Repository: http://dx.doi.org/10.5061/dryad.1rs10.

## Supporting Information

Appendix S1 Survival models and analyses.

Appendix S2 Strength of selection model.

Appendix S3 Altered blood-feeding and capacity to transmit disease.
