# Supplementary material for: Reduced survival and reproductive success generates selection pressure for the dengue mosquito Aedes aegypti to evolve resistance against infection by the microsporidian parasite Vavraia culicis
Source: Evol Appl. 2014 Feb 7;7(4):468–79. doi: 10.1111/eva.12144 (PMC4001445; doi:10.1111/eva.12144)
Supplement: Appendix S1 — Survival models and analyses. [file eva0007-0468-sd1.doc]

**Appendix 1**

This Appendix file describes;

(i) the general approach used to assess the survival of adult female mosquitoes,

(ii) the best fit survival models comparing different treatments in Experiment II, and

(iii) the best fit survival models for females of Experiment I and those of the 'single-blood-meal' and 'multiple-blood-meal' treatments of Experiment II*.*

*(i) The approach used to estimate adult female mosquito survival*

The survival of adult female mosquitoes in the three weeks following the first opportunity to blood feed was estimated using the Weibull survival function

$$S\left(t\right)=\exp\left\{-\left(\lambda t\right)^{\alpha}\right\}$$

where survival to time *t*, *S*(*t*), depended on the hazard function

$$\lambda\left(t\right)=\alpha\lambda\left(\lambda t\right)^{\alpha-1}$$

for *λ* > 0 and *α* > 0.

The negative log likelihood of this function for right-censored data

$$-\ln L=-\sum\left\{d_{i}\left[\ln\lambda_{i}+\ln\alpha_{i}+\left(\alpha_{i}-1\right)\ln\left(\lambda_{i}t\right)\right]-\left(\lambda_{i}t\right)^{\alpha_{i}}\right\}$$

was fitted using the non-linear platform of JMP where the index *d_i_* was defined by whether female *i* was censored or not; censored females (*d* = 1) were those still alive at the end of either experiment or females removed from cages during Experiment II because they had not taken a blood meal when given the opportunity to do so, uncensored females (*d* = 0) were those that died during the experiments.

Estimates of *λ_i_* and *α_i_* were based on linear equations of the form

$$\lambda_{i}=\exp\left\{-\left(\sum b_{i}x_{ij}\right)\right\}$$

and

$$\alpha_{i}=\sum a_{j}x_{ij}$$

where $j=1,2,\cdots,k$ were the number of explanatory variables in the model.

These variables were determined by experimental parameters, e.g. the infection status of female *i* or the treatment in which she participated, or by environmental parameters where infected and uninfected females shared a common environment, e.g. being in the same experiment, treatment within experiment, or cage within treatment.

The results of different models were classified according to Akaike’s information criterion

$$AIC=2k+n\ln\left(\frac{sse}{n}\right)$$

where *k* was the number of parameters estimated, *n* the total number of observations, *sse* the error sums of squares estimated by JMP.

The strength of competing models to explain the data were assessed with a weighted index of their *AIC* values

where individual *AIC*s were compared to that of the minimum *AIC* (= Δ).

*(ii) The effect of treatment conditions on the survival of adult females in Experiment II*

Full models estimating *λ_i_* and *α_i_* of female *i* in Experiment II were of the form

$$\lambda_{i}=\exp\left[-\left(b_{0}+b_{1}x_{i1}+b_{2}x_{i2}+b_{3}x_{i3}+b_{4}x_{i4}\right)\right]$$

and

$$\alpha_{i}=a_{0}+a_{1}x_{i1}+a_{2}x_{i2}+a_{3}x_{i3}+a_{4}x_{i4}$$

where *b_0_* and *a_0_* were common to all females, *b_1_* and *a_1_* estimated the effect of infection for female *i*, *b_2_* and *a_2_* estimated the effect of the treatment she experienced, *b_3_* and *a_3_* estimated the interaction between infection and treatment, while *b_4_* and *a_4_* estimated the environmental effects associated with females sharing individual cages within treatments. When the survival of female *i* was modelled following an exponential distribution, *a_0_* and *a_i1_* were set to values of 1 and 0, respectively.

The 10 best models found according to the *AIC_w_* index are shown in rank order in Table A1.

Table A1. Rank order of best models estimating the survival of females in the different treatments of Experiment II

| Model | Parameters estimated | *k* | *sse* | *AIC* | Δ | *AIC_w_* |
| --- | --- | --- | --- | --- | --- | --- |
| 1 | *b_0_, b_1_, b_2_, a_1_* | 4 | 867.00 | 241.8 | 0.0 | 15.1 |
| 2 | *b_0_, b_1_, a_1_* | 3 | 870.65 | 242.2 | 0.4 | 12.2 |
| 3 | *b_0_, b_1_, b_2_, a_0_* | 4 | 868.06 | 241.8 | 0.7 | 10.6 |
| 4 | *b_0_, b_1_, a_0_* | 3 | 872.01 | 243.1 | 1.3 | 7.8 |
| 5 | *b_0_, b_1_, a_1_, a_2_* | 4 | 869.26 | 243.3 | 1.5 | 7.1 |
| 6 | *b_0_, b_1_, b_2_, a_0_, a_1_* | 5 | 866.53 | 243.4 | 1.7 | 6.5 |
| 7 | *b_0_, b_1_, b_2_, a_1_* | 5 | 866.88 | 243.7 | 1.9 | 5.8 |
| 8 | *b_0_, b_1_, b_2_, b_3_, a_1_* | 5 | 866.89 | 243.7 | 1.9 | 5.8 |
| 9 | *b_0_, b_1_, b_2_, a_1_, a_2_* | 5 | 866.94 | 243.7 | 2.0 | 5.7 |
| 10 | *b_0_, b_1_, b_2_, a_1_, a_4_* | 6 | 866.94 | 243.7 | 2.2 | 5.1 |

The best fit survival model found the estimates of *λ_i_* and *α_i_* shared by all females in Experiment II (*b_0_*, *a_0_*) were significantly influenced by the infection status of female *i* and that of the treatment she experienced (*b_2_*).

The estimates of *λ_i_* and *α_i_* for the best fit model for uninfected females were

$$\lambda_{i}=\exp\left[-\left(b_{0}+b_{1}+b_{2}\right)\right]=\exp\left[-\left(3.964+0.659+b_{2}\right)\right]$$

$$\alpha_{i}=a_{0}=1$$

and those of infected females were,

$$\lambda_{i}=\exp\left[-\left(b_{0}+b_{1}+b_{2}\right)\right]=\exp\left[-\left(3.964-0.659+b_{2}\right)\right]$$

$$\alpha_{i}=a_{0}+a_{1}=1+0.481$$

respectively, where *b_2_* = -0.169 for females from the 'sugar-only' treatment and *b_2_* = 0.169 for females from the 'single-blood-meal' and 'multiple-blood-meal' treatments.

Thus, the overall survival of uninfected females in Experiment II was best described by a constant exponential decline, while that of infected females increased with their age, to which an additional increase in mortality could be added for females that did not take a blood meal.

*(iii) The best fit survival models for females of Experiment I and those of the 'single-blood-meal' and 'multiple-blood-meal' treatments of Experiment II.*

The full models in these analyses allowed for female *i* sharing the same experiment, treatment within experiment, cage within treatment and/or experiment, with other females and the experimental effects of treatment (within Experiment II), infection and their interactions.

The 10 best models found according to the *AIC_w_* index are shown in rank order in Table A2.

Table A2. Rank order of best models estimating the survival of females from Experiment I and the 'single-blood-meal' and 'multiple-blood-meal' treatments of Experiment II

| Model | Parameters estimated | *k* | *sse* | *AIC* | Δ | *AIC_w_* |
| --- | --- | --- | --- | --- | --- | --- |
| 1 | *b_0_, b_1_, a_1_* | 3 | 1066.26 | 267.9 | 0.0 | 33.8 |
| 2 | *b_0_, b_1_, a_1_, a_5_* | 4 | 1065.26 | 269.2 | 1.3 | 17.7 |
| 3 | *b_0_, b_1_, a_0_, a_1_* | 4 | 1066.19 | 269.9 | 2.0 | 12.8 |
| 4 | *b_0_, b_1_, a_1_, b_5_* | 4 | 1066.21 | 269.9 | 2.0 | 12.7 |
| 5 | *b_0_, b_1_* | 2 | 1072.73 | 270.5 | 2.6 | 9.4 |
| 6 | *b_0_, b_1_, b_5_, a_1_, a_5_* | 5 | 1065.03 | 271.1 | 3.1 | 7.1 |
| 7 | *b_0_, b_1_, b_5_, b_6_, a_1_, a_5_* | 6 | 1064.78 | 272.9 | 5.0 | 2.8 |
| 8 | *b_0_, b_1_, b_5_, a_1_, a_5_, a_6_* | 6 | 1065.02 | 273.1 | 5.1 | 2.6 |
| 9 | *b_0_, b_1_, b_5_, b_6_, a_1_, a_5_, a_6_* | 7 | 1064.64 | 274.8 | 6.9 | 1.1 |
| 10 | *b_0_* | 1 | 1104.62 | 290.5 | 22.6 | 0.0 |

The estimates of *λ_i_* and *α_i_* for the best fit model for uninfected females were

$$\lambda_{i}=\exp\left[-\left(b_{0}+b_{1}\right)\right]=\exp\left[-\left(4.158+0.653\right)\right]$$

$$\alpha_{i}=a_{0}=1$$

and those of infected females were,

$$\lambda_{i}=\exp\left[-\left(b_{0}+b_{1}\right)\right]=\exp\left[-\left(4.158-0.653\right)\right]$$

$$\alpha_{i}=a_{0}+a_{1}=1+0.345$$

where *b_0_* and *a_0_* were shared for all females and *b_1_*, *a_1_* estimated the effects of infection for female *i*.

Thus, the best model described the survival of uninfected females as following a constant exponential decline (*α_i_* = 1), while that of infected females increased with their age (*α_i_* = 1.345).

The second best fit model included a term (*a_5_*) allowing estimates of *α_i_* to vary for infected females between the two experiments; Experiment I, *α_i_* = 1.262, Experiment II, *α_i_* = 1.404. The third best model was similar to the best fit model except that *a_0_* was estimated, rather than being set to 1; with the estimated value being 0.959 with upper and lower 95% confidence intervals of 0.699 and 1.299, respectively.

The other variables estimated in Table A2 allowed for variation in *λ_i_* due to the environmental effect of experiment (*b_5_*), or interactions between experiment and infection for female *i* (*b_6_*, *a_6_*). None of the leading models involved estimates allowing for variation among individual cages within each experiment.
