# Supplementary material for: Reduced survival and reproductive success generates selection pressure for the dengue mosquito Aedes aegypti to evolve resistance against infection by the microsporidian parasite Vavraia culicis
Source: Evol Appl. 2014 Feb 7;7(4):468–79. doi: 10.1111/eva.12144 (PMC4001445; doi:10.1111/eva.12144)
Supplement: Appendix S2 — Strength of selection model. [file eva0007-0468-sd2.doc]

**Appendix 2**

In this appendix file we present a model predicting the strength of selection experienced by *V. culicis*-infected mosquitoes as a function of larval developmental times.

Larvae are assumed to be infected by *V. culicis* within 24 h of hatching. The number of spores harboured by infected individuals on a given day, *Sp*(*t*), increases as

$$Sp\left(t\right)=if\left\{\begin{matrix} t<c & 0 \\ t\geq c & \left(\lambda_{sp}\left[t-c\right]\right)^{\alpha_{sp}} \end{matrix}\right.$$

where *c* represents the incubation period in days of *V. culicis* prior to spore production, with the number of spores subsequently produced determined by the shape and scale parameters, *α_sp_* and *λ_sp_*, respectively. The accumulating risk of mortality for infected females due to the accumulation of spores, *H*(*t*), is

$$H_{i}\left(t\right)=if\left\{\begin{matrix} t<c & 0 \\ t\geq c & k_{i}Sp\left(t\right) \end{matrix}\right.$$

where the index *i* designates the juvenile (*juv*) or adult (*ad*) stages of development and the constant *k* scales the risk of mortality for each stage. The survival of infected adult females, relative to uninfected adult females, *S_inf_*(*t*), is thus

$$S_{inf}\left(t\right)=\exp\left\{-H_{juv}\left(t_{a}\right)\right\}\cdot\exp\left\{-H_{ad}\left(t-t_{a}\right)\right\}$$

where *t_a_* denotes age at emergence in days. The first term describes the relative survival of infected females to adulthood, while the second term describes their relative survival as adults for *t* > *t_a_*. As the relative survival of infected females is being estimated, it is independent of the common background mortality experienced by infected and uninfected females in either developmental stage. The complement of this function, 1 - *S_inf_*(*t*), estimates the coefficient of selection against infected females based on their relative survival to time *t*.
