# Supplementary material for: Reduced survival and reproductive success generates selection pressure for the dengue mosquito Aedes aegypti to evolve resistance against infection by the microsporidian parasite Vavraia culicis
Source: Evol Appl. 2014 Feb 7;7(4):468–79. doi: 10.1111/eva.12144 (PMC4001445; doi:10.1111/eva.12144)
Supplement: Appendix S3 — Altered blood-feeding and capacity to transmit disease. [file eva0007-0468-sd3.doc]

**Appendix 3**

This appendix file presents calculations for the influence of *Vavraia culicis* on the capacity of female mosquitoes to contract an agent of human disease in their first gonotrophic cycle and to survive long enough to subsequently transmit it.

These calculations are described by the following inequality composed of three parts;

(i) the relative probability of females taking an infected blood meal during their first gonotrophic cycle when infected with *V. culicis* or not,

(ii) the relative probability of *V. culicis*-infected vs. -uninfected females surviving blood-feeding events during the first and subsequent gonotrophic cycles in a period equal to the extrinsic incubation period (EIP) of the human pathogen, and

(iii) the relative survival of *V. culicis*-infected vs. -uninfected females in the period equal to the EIP of the human pathogen, independent of their blood-feeding behaviour (i.e. relative survival as calculated in the main text).

If the inequality > 1, *V. culicis*-infected females are more likely to become infected in their first gonotrophic cycle and survive the EIP of the human pathogen, i.e. they are potentially relatively better vectors of human disease.

$$\frac{1-\left(1-a\right)^{b_{2}}}{1-\left(1-a\right)^{b_{1}}}\cdot\frac{d^{b_{2}G_{2}}}{d^{b_{1}G_{1}}}\cdot\exp\left[-\left(\lambda e\right)^{\alpha}\right]>1$$

In the first term of the expression, *a* is the prevalence of disease in the human population, *b_2_* and *b_1_* are the respective number of bites females infected or uninfected with *V. culicis* take to complete a gonotrophic cycle.

In the second term of the expression *d* is the probability a female survives taking a blood meal, *G_2_* and *G_1_* are the respective number of gonotrophic cycles females infected or uninfected with *V. culicis* complete between the start of the first gonotrophic cycle and the EIP of the human pathogen.

The third tem of the expression is the relative survival of *V. culicis*-infected females in the period between the start of the 1st gonotrophic cycle and the EIP of the human pathogen, *e*, as determined by the shape and scale parameters of the Weibull survival function.

It is assumed females take a fixed volume of blood *v* at each meal and need a total *V* of blood to complete a gonotrophic cycle. The number of blood meals required to complete a gonotrophic cycle is then,

$$b_{i}=\frac{V_{i}}{v_{i}}$$

and the time take to complete a single gonotrophic cycle

$$g_{i}=r_{i}b_{i}+m_{i}$$

where *r* is the daily biting rate of females and *m* is the time required for maturation and oviposition of eggs.

The number of gonotrophic cycles completed during the EIP of an agent of human disease is then,

$$G_{i}=\frac{e}{g_{i}}=\frac{e}{r_{i}b_{i}+m_{i}}=\frac{ev_{i}}{r_{i}V_{i}+m_{i}v_{i}}$$

These calculations provide an estimate for the potential of female mosquitoes infected with *V. culicis* to transmit human disease relative to females not infected with *V. culicis*. This is a relative estimate and does not include the background mortality experienced by both infected and uninfected females.

Worked example

The figure below shows the probability of a female uninfected with *V. culicis* becoming infected with an agent of human disease in her first gonotrophic cycle and surviving its EIP as a function of prevalence of disease in human population (*a*) and the probability a female survives a blood-feeding event (*d*).

The values used in calculating this figure were;

*V_1_* = 1.0 (= total volume of blood per gonotrophic cycle)

*b_1_* = 0.85 (= biting rate day^-1^)

*v_1_* = 1.0 (= volume of blood taken per blood meal)

*m_1_* = 2 (= time in days to digest/oviposit eggs)

EIP = 10 days

Thus in this estimate it is assumed that when females are not infected by *V. culicis*, they take all the blood they need to complete a gonotrophic cycle in a single blood-feeding event. The biting rate used (*b_1_*) approximates that observed in Experiment I of this study. Once sufficient blood has been acquired, it is assumed females will require an additional two days to mature and oviposit a clutch of eggs. The EIP used is towards the lower bound of estimates for the EIP of the dengue virus.

With these values, the time taken for a female to take a volume *V* of blood equals 1.176 days and the number of gonotrophic cycles completed by 10 days is 3.148.

The figure below is based on the same calculation as above, using values for females infected with *V. culicis*

The values used in calculating this figure were;

*V_2_* = 1.0 (= total volume of blood per gonotrophic cycle)

*b_2_* = 0.65 (= biting rate day^-1^)

*v_2_* = 0.75 (= volume of blood taken per blood meal)

*m_2_* = 2 (= time in days to digest/oviposit eggs)

EIP = 10 days

Relative survival = exp[-(0.025 x 10)^1.571^]

In this case, it is assumed *V. culicis*-infected females require the same total volume of blood *V* to mature a clutch of eggs as females not infected by *V. culicis*. However, the daily biting rate and the relative volume of blood taken per blood-feeding event are reduced to values observed for *V. culicis*-infected females in Experiment I of this study. The relative survival of *V. culicis*-infected females also takes on values estimated in the main text.

With these values the time taken for females infected with *V. culicis* to take a volume *V* of blood equals 2.051 days and the number of gonotrophic cycles they complete in 10 days is 2.468.

The following figure shows the relative probabilities of *V. culicis*-infected vs. -uninfected females contracting an agent of human disease in her first gonotrophic cycle and surviving the EIP of the agent from the two figures above,

The darker region shows conditions in which females infected with *V. culicis* are estimated as being relatively more likely to contract an agent of human disease in their first gonotrophic cycle and survive its EIP, given the prevalence of infection in the human population (*a*) and the probability a female survives a blood-feeding event (*d*).
